# A Modified Prandtl–Ishlinskii Hysteresis Model for Modeling and Compensating Asymmetric Hysteresis of Piezo-Actuated Flexure-Based Systems

**DOI:** 10.3390/s22228763

**Published:** 2022-11-13

**Authors:** Chao Zhou, Meng Yuan, Chen Feng, Wei Tech Ang

**Affiliations:** School of Mechanical and Aerospace Engineering, Nanyang Technological University, Singapore 639798, Singapore

**Keywords:** asymmetric hysteresis, inverse hysteresis compensation, piezoelectric actuators, piezo-actuated flexure-based system

## Abstract

Piezo-actuated flexure-based systems are widely used in applications with high accuracy requirements, but the intrinsic hysteresis has a detrimental effect on the performance which should be compensated. Conventional models were presented to model this undesired effect using additional dead-zone operators. This paper presents a new approach using two sets of operators with a distributed compensator to model and compensate for the asymmetric system hysteresis based on inversion calculation with a simplified digitized representation. The experimental results validate the effectiveness of the proposed model in modeling and compensating the asymmetric system hysteresis.

## 1. Introduction

Piezoelectric actuators are ideal for micromanipulation due to their fast response with high repeatability, but they often have a limited stroke. The flexure mechanism are often used together with the piezoelectric actuators to amplify their stroke with no backlash. The piezo-actuated flexure-based systems are used in precise fabrication [1,2,3], autofocus optical systems [4,5,6], microsurgical robots [7,8,9], and other applications [10,11,12]. One of the biggest challenges while using a piezo-actuated flexure-based system is to deal with the undesired system hysteresis.

**Figure 1:**
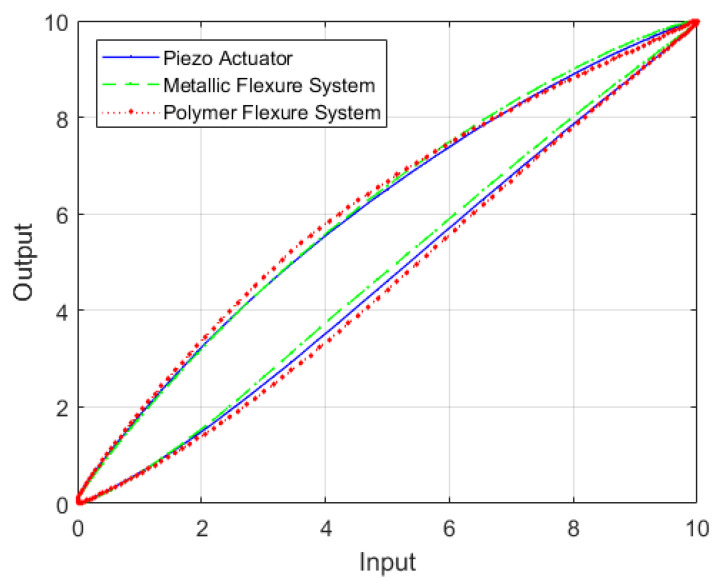
The hysteresis behavior of a piezoelectric actuator, a piezo-actuated metallic flexure-based system and a piezo-actuated polymer flexure-based system after normalization.

Hysteresis is a very complex phenomenon with different possible causes. Hysteresis may arise from material properties [13,14], mechanisms [15,16,17], and others [18,19]. The hysteresis behavior of an actuator is compared to that of piezo-actuated different material built flexure-based systems after normalization, as shown in Figure 1. The figure shows that the system hysteresis can be a combination of the flexure mechanism hysteresis and the piezoelectric actuator hysteresis. The hysteresis of the flexure mechanism may add to the complexity of the system hysteresis.

Various models were proposed to describe the hysteresis. The phenomenology-based models are classified into roughly three groups [20]: differential-based models, operator-based models and the intelligent models. The Duhem [21,22] model and the Bouc-wen [23,24] model are two popular differential-based models that use differential equations to model the hysteresis behavior. It is convenient to design controllers combined with dynamic models due to the compact structure of differential-based models, but their inverse model construction as well as the parameters identification are often difficult. Intelligent models are very popular in recent years and can also be used to model the hysteresis. Some examples are Support Vector Machine (SVM) [25,26], the neural networks [27,28], and others. These models have good performance in some cases, but these models are often difficult to be explained using mathematics and may be not interpretable.

The construction of the operator-based models are often easier, among the various operator-based models the Prandtl–Ishlinskii (PI) [29] model is widely adopted. The Prandtl–Ishlinskii (PI) model can be easily implemented using an analytical inversion [30]. Ang [31] proposed a linear relationship between the value of the operators and the actuation rate. Tan [32] modified the classical model for hysteresis behavior with a negative gradient. The asymmetric hysteresis behavior has also been studied: Kuhnen [33] introduced dead-zone operators to deal with the memory-free asymmetric hysteresis behavior. Gu [34] proposed to use a polynomial input function instead of the linear function. Mohammad [29,35] presented a generalized Prandtl–Ishlinskii (GPI) model to characterize the asymmetric hysteresis behavior, and the inversion of an extended generalized Prandtl–Ishlinskii (EGPI) Hysteresis model was described by Zhang [36]. However, the envelope function brings challenges to parameter identifications. Sun [37] proposed an extended unparallel Prandtl–Ishlinskii (EUPI) model while only the left descending edge of a CPI operator can freely tilt.

In this paper, a dual-operators-based modified Prandtl–Ishlinskii (DPI) model is proposed to describe and compensate for the asymmetric system hysteresis, which is an extension of our previous work [38], where the highly asymmetric hysteresis behavior of the piezo-actuated flexure-based system is not discussed. In this work, the proposed framework can capture the details of the asymmetric system hysteresis. The feedforward compensator can control the piezo-actuated flexure-based system with high accuracy. The proposed model and its inverse hysteresis compensator are validated with modeling and control experimental results.

The rest of this paper is organized as follows. Section 2 presents the DPI model and its analytical inversion. The validation experimental results are described in Section 3. Section 4 and Section 5 cover the discussion and the conclusion, respectively.

## 2. Hysteresis Mathematical Model

This section describes a digitized representation and the proposed DPI model. The inverse DPI model and the link between the classical and the digitized representation are also described.

### 2.1. Digitized Representation

Here, we adopt the similar digitized representation of the classical Prandtl–Ishlinskii model in [38]. The state of the classical PI hysteresis model with *N* operators is represented by a binary number *B* with *N* digits, as shown in following equation:

(1) $$B=\{b_{1},\ldots ,b_{i},\ldots ,b_{N}\},$$

where i=1,…,N and $b_{i}=\left\{0,1\right\}$. The system input and output with state *B* are represented with X(B)∈R and Y(B)∈R, respectively.

Each fundamental operator, $b_{i}$, has a paired properties: $\left(\Delta X_{i},\Delta Y_{i}\right)$, which stands for the changes in system input and output, respectively. The values of $\Delta X_{i}\in R$ and $\Delta Y_{i}\in R$ are fixed for particular system. The fundamental operators are represented with *H* as:

(2) $$H=\left[\begin{array}{ccccc} \Delta X_{1} & \ldots & \Delta X_{i} & \ldots & \Delta X_{N}\\ \Delta Y_{1} & \ldots & \Delta Y_{i} & \ldots & \Delta Y_{N} \end{array}\right].$$

The change of operator $b_{i}$ follows the below proposition:

The binary state of a fundamental operator $b_{i}$ can be changed from 0 to 1 during expansion, and from 1 to 0 during contraction. The most significant digit is $b_{n}$ while $b_{1}$ is the least significant digit. The change of the state is always from the least significant digit. Denote the altered binary state of $b_{i}$ be $b_{i}^{+}$. When the binary state of a fundamental operator $b_{i}$ is changed from 0 to 1 during expansion, the change of the system input and output will then change according to its paired properties using the following equations:

(3) $$X\left(B_{i^{+}}\right)=X\left(B\right)+\Delta X_{i},$$

(4) $$Y\left(B_{i^{+}}\right)=Y\left(B\right)+\Delta Y_{i},$$

where $B_{i^{+}}≜\left\{b_{N},\ldots ,b_{i}^{+},\ldots ,b_{1}\right\}$.

When the binary state of a fundamental operator $b_{i}$ is changed from 1 to 0 during contraction, the change of the system input and output will then change according to its paired properties using the following equations:

(5) $$X\left(B_{i^{+}}\right)=X\left(B\right)-\Delta X_{i},$$

(6) $$Y\left(B_{i^{+}}\right)=Y\left(B\right)-\Delta Y_{i},$$

where $B_{i^{+}}≜\left\{b_{N},\ldots ,b_{i}^{+},\ldots ,b_{1}\right\}$.

**Figure 2:**
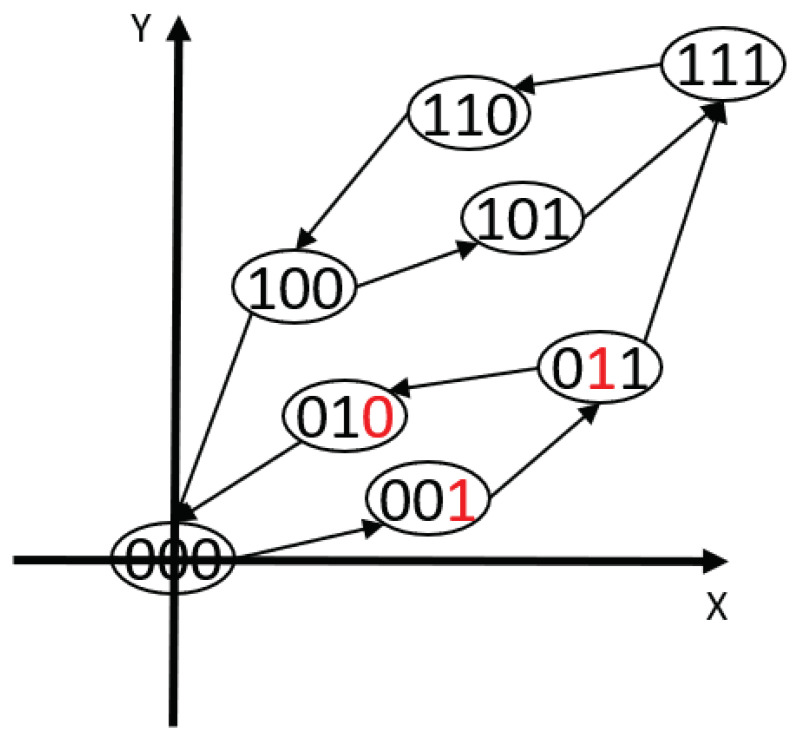
A digitized representation of the classical PI hysteresis model with three operators.

A simple example with 3 operators can be shown in Figure 2. The above equations can be further simplified, when the binary state of a fundamental operator $b_{i}$ is changed, the change of the system input and output will then change according to its paired properties using the following equations:

(7) $$X\left(B_{i^{+}}\right)=X\left(B\right)+\Delta X_{i}^{1-b_{i}}\cdot {\left(-\Delta X_{i}\right)}^{b_{i}},$$

(8) $$Y\left(B_{i^{+}}\right)=Y\left(B\right)+\Delta Y_{i}^{1-b_{i}}\cdot {\left(-\Delta Y_{i}\right)}^{b_{i}},$$

where $B_{i^{+}}≜\left\{b_{N},\ldots ,b_{i}^{+},\ldots ,b_{1}\right\}$.

### 2.2. DPI Model

The classical PI model requires the hysteresis behavior to be symmetric, this limitation is mainly due to the one-paired property of the play operator for modeling the expansion curve and the contraction curve. In this section, the DPI model for modeling and compensating the asymmetric system hysteresis is presented. The proposed DPI model applies two sets of operators for modeling the expansion curve and the contraction curve respectively with a similar digitized representation.

We used the same representation as (1) to represent the state of the system using the DPI model with *N* fundamental operators.

**Figure 3:**
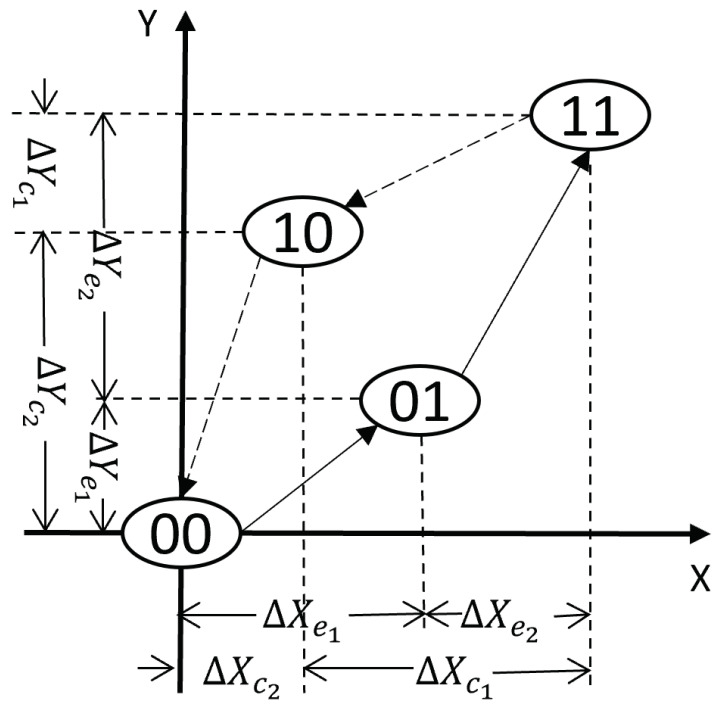
DPI model with two fundamental operators.

Compared to the previous one, each digit $b_{i}$ in (1) has two paired properties: $\left(\Delta X_{e_{i}},\Delta Y_{e_{i}}\right)$, and $\left(\Delta X_{c_{i}},\Delta Y_{c_{i}}\right)$. The two paired properties are used for modeling the expansion curve and contraction curve respectively. The change of system input and output during expansion are represented with $\left(\Delta X_{e_{i}},\Delta Y_{e_{i}}\right)$, while the changes of the system during contraction are represented with $\left(\Delta X_{c_{i}},\Delta Y_{c_{i}}\right)$. The properties of the DPI model with two fundamental operators are illustrated in Figure 3. The properties $H_{e}$ and $H_{c}$ of the DPI model with *N* elementary operators are expressed in the following equations:

(9) $$H_{e}=\left[\begin{array}{cccc} \Delta X_{e_{1}} & \ldots \Delta X_{e_{i}} & \ldots & \Delta X_{e_{N}}\\ \Delta Y_{e_{1}} & \ldots \Delta Y_{e_{i}} & \ldots & \Delta Y_{e_{N}} \end{array}\right],$$

where $\Delta X_{e_{i}}\geq 0$.

(10) $$H_{c}=\left[\begin{array}{cccc} \Delta X_{c_{1}} & \ldots \Delta X_{c_{i}} & \ldots & \Delta X_{c_{N}}\\ \Delta Y_{c_{1}} & \ldots \Delta Y_{c_{i}} & \ldots & \Delta Y_{c_{N}} \end{array}\right],$$

where $\Delta X_{c_{i}}<0$.

Similarly, the change of the system input and output will change according to its paired properties using the following equations:

(11) $$X\left(B_{i^{+}}\right)=X\left(B\right)+\Delta X_{e_{i}}^{1-b_{i}}\cdot \Delta X_{c_{i}}^{b_{i}}.$$

(12) $$Y\left(B_{i^{+}}\right)=Y\left(B\right)+\Delta Y_{e_{i}}^{1-b_{i}}\cdot \Delta Y_{c_{i}}^{b_{i}}.$$

The sum of the expansion operators may not equal to the sum of the contraction operators, and this may lead to drifting of the proposed model in some cases. With a repetitive input, the drifting will accumulate and the output of the model may reach infinity, which may lead to the failure of the proposed model. For example, when $Y(b_{N},\ldots ,b_{2},b_{1})$ changes to $Y(b_{N},\ldots ,b_{2}^{+},b_{1}^{+})$, and finally reaches $\bar{Y}\left(b_{N},\ldots ,b_{2},b_{1}\right)$:

(13) $$\bar{Y}\left(B\right)=Y\left(B\right)+\Delta Y_{e_{1}}+\Delta Y_{e_{2}}+\Delta Y_{c_{1}}+\Delta Y_{c_{2}}.$$

The expansion and contraction properties may not equal:

(14) $$\Delta Y_{e_{1}}+\Delta Y_{e_{2}}+\Delta Y_{c_{1}}+\Delta Y_{c_{2}}\neq 0,$$

and

(15) $$\bar{Y}\left(B\right)\neq Y\left(B\right).$$

We denote $B_{a}$ as the value of state with all elements as *a*, i.e., $b_{i}=a,i=Z_{\left[1,N\right]}$, where $Z_{\left[a,b\right]}$ is the set of integer numbers in range [a,b]. A compensator $T_{c}\left(B\right)$ is proposed using the following equation when the system contracts after a turning point:

(16) $$T_{c}\left(B\right)=Y\left(B\right)-Y\left(B_{0}\right)+\sum_{i=1}^{N}b_{i}\cdot \Delta Y_{c_{i}}.$$

The contraction paired properties are then updated at the turning point:

(17) $$\Delta {\hat{Y}}_{c_{i}}=\Delta Y_{c_{i}}+T_{c}\left(B\right)\cdot f_{c}\left(i\right),$$

where $f_{c}\left(i\right)$ is a distribution with a sum of 1,

(18) $$f_{c}\left(i\right)=\frac{\Delta Y_{c_{i}}}{\sum_{i=1}^{N}b_{i}\cdot \Delta Y_{c_{i}}}.$$

Similarly, a compensator $T_{e}\left(B\right)$ is proposed using the following equation when the system expands after a turning point:

(19) $$T_{e}\left(B\right)=Y\left(B\right)-Y\left(B_{1}\right)+\sum_{i=1}^{N}\left(1-b_{i}\right)\cdot \Delta Y_{e_{i}}.$$

The expansion paired properties are then updated at the turning point:

(20) $$\Delta {\hat{Y}}_{e_{i}}=\Delta Y_{e_{i}}+T_{e}\left(B\right)\cdot f_{e}\left(i\right),$$

where $f_{e}$ is a distribution with a sum of 1,

(21) $$f_{e}\left(i\right)=\frac{\Delta Y_{e_{i}}}{\sum_{i=1}^{N}\left(1-b_{i}\right)\cdot \Delta Y_{e_{i}}}.$$

At turning points, the value of operators are updated with an addition of a distributed compensator to avoid drifting. An equal weight distribution is chosen so that the value of each operator is not modified too much. When the proposed method is applied to other systems with hysteresis, different distribution strategies can be chosen subject to the observations of the hysteresis behavior. An assumption is also made that $X(B_{0})$ and $X(B_{1})$ are fixed values. This can be further modified with other systems by setting alternative stationary values or boundaries to help calculate the value of the compensator.

### 2.3. Inverse DPI Model

Similar to the previous work in [38], the inverse compensator of the proposed model can be constructed by simply exchanging $\Delta X_{e_{i}}$ with $\Delta Y_{e_{i}}$, and $\Delta X_{c_{i}}$ with $\Delta Y_{c_{i}}$ within the two paired properties, the properties of the inverse model can be obtained using the following equations:

(22) $$\overset{´}{H_{e}}=\left[\begin{array}{cc} 0 & 1\\ 1 & 0 \end{array}\right]H_{e}.$$

(23) $$\overset{´}{H_{c}}=\left[\begin{array}{cc} 0 & 1\\ 1 & 0 \end{array}\right]H_{c}.$$

The process of calculation in the system identification is simplified with the proposed method. The order of computation complexity can be reduced greatly to O(N) for our method.

### 2.4. Comparison of the Inverse Model Parameters Using the Digitized and the Classical Representation

The inverse model $\Delta X_{i}^{{}^{\prime }}$, $\Delta Y_{i}^{{}^{\prime }}$ using the digitized representation can be expressed in the following equations [38]:

(24) $$\Delta X_{i}^{{}^{\prime }}=2\left(r_{i+1}-r_{i}\right)\cdot \sum_{j=0}^{i}w_{j}.$$

(25) $$\Delta Y_{i}^{{}^{\prime }}=2\left(r_{i+1}-r_{i}\right).$$

The inverse model parameters $r_{i}^{{}^{\prime }}$ with the classical representation can be calculated [32] by:

(26) $$r_{0}^{{}^{\prime }}=0.$$

(27) $$r_{i}^{{}^{\prime }}=\sum_{j=0}^{i}w_{j}\left(r_{i}-r_{j}\right),i=1,\ldots ,N.$$

(28) $$r_{i+1}^{{}^{\prime }}-r_{i}^{{}^{\prime }}=\left(r_{i+1}-r_{i}\right)\cdot \sum_{j=0}^{i}w_{j},i=1,\ldots ,N.$$

From (24), we can get:

(29) $$\Delta X_{i}^{{}^{\prime }}=2\left(r_{i+1}^{{}^{\prime }}-r_{i}^{{}^{\prime }}\right)=2\left(r_{i+1}-r_{i}\right)\cdot \sum_{j=0}^{i}w_{j}.$$

The inverse model parameters $w_{i}^{{}^{\prime }}$ with the classical representation can be calculated [32] by:

(30) $$w_{0}^{{}^{\prime }}=\frac{1}{w_{0}}.$$

(31) $$w_{i}^{{}^{\prime }}=\frac{-w_{i}}{\left(\sum_{j=0}^{i}w_{j}\right)\left(\sum_{j=0}^{i-1}w_{j}\right)},i=1,\ldots ,N.$$

Alternatively, we can calculate the $w_{i}^{{}^{\prime }}$ using the digitized representation:

(32) $$\begin{array}{cc} w_{i}^{{}^{\prime }} & =\frac{\Delta Y_{i}^{{}^{\prime }}}{\Delta X_{i}^{{}^{\prime }}}-\frac{\Delta Y_{i-1}^{{}^{\prime }}}{\Delta X_{i-1}^{{}^{\prime }}}\\ \; & =\frac{-w_{i}}{\left(\sum_{j=0}^{i}w_{j}\right)\left(\sum_{j=0}^{i-1}w_{j}\right)},\;i=1,\ldots ,N. \end{array}$$

The equations above show that the inverse model parameters are the same using the digitized and the classical representation. Moreover, the calculation complexity can be reduced with the proposed digitized representation.

## 3. Experimental Results

### 3.1. Experimental Setup

**Figure 4:**
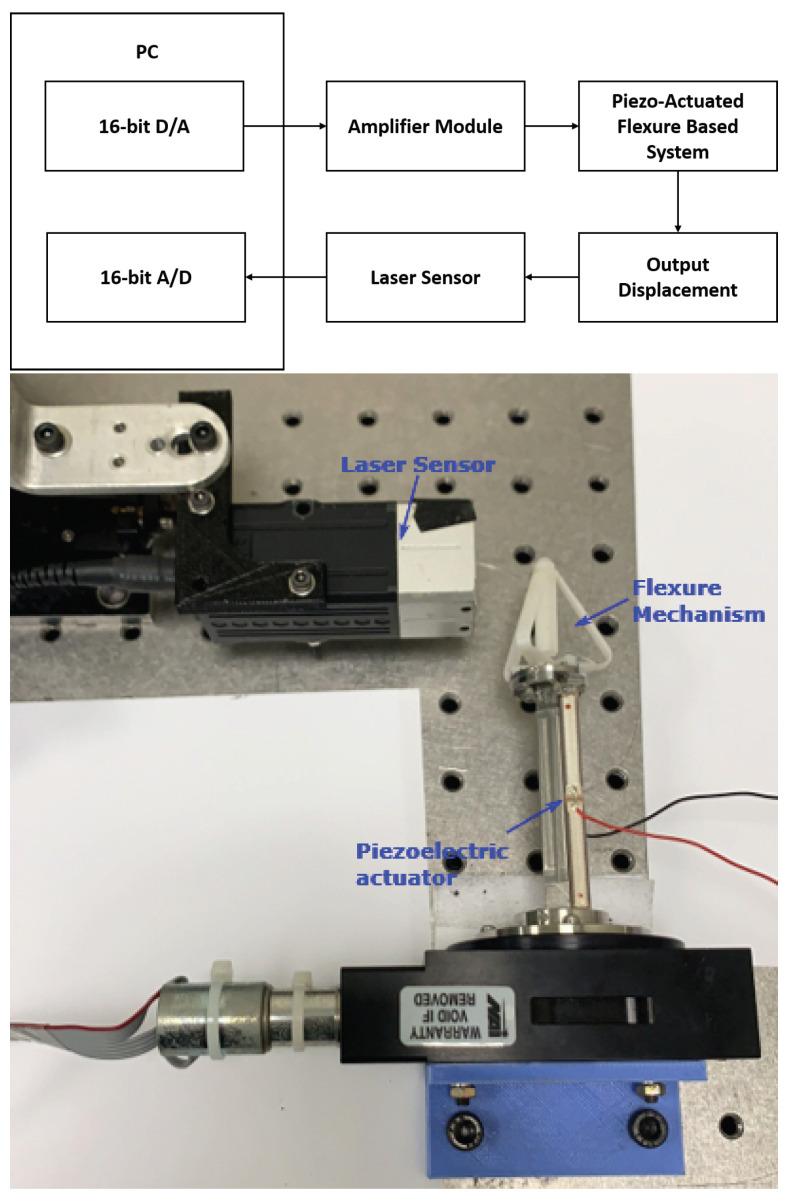
Experimental setup.

In this section, the same 16-bit D/A card, actuator, amplifier, sensor, 16-bit A/D card used in [38] is chosen. The asymmetric system hysteresis behavior of the piezo-actuated flexure-based mechanism is studied for experimental validation, as shown in Figure 4. The experiments are performed in an air-conditioned room so that the temperature factor can be neglected.

In this section, the system hysteresis behavior is modeled using the PI, modified PI model with dead zone operators (MPI), and the DPI model, respectively. The inverse model is then identified to compensate for the system hysteresis under fixed amplitude and changing amplitude using the PI, MPI, and DPI model, respectively. A brief description of the experimental setup is first given.

### 3.2. Modeling Results

**Figure 5:**
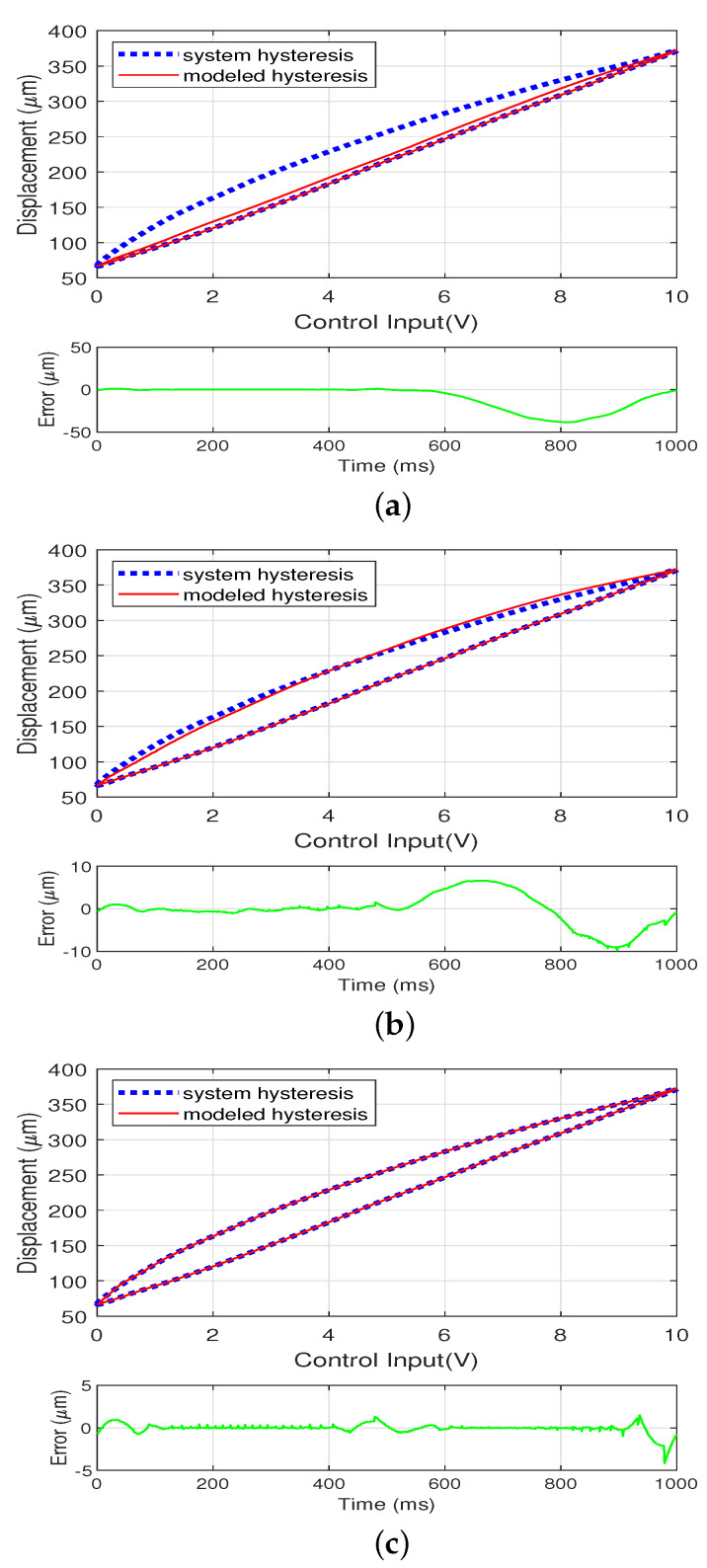
Modeling results for the system hysteresis using the PI, MPI and the DPI model, respectively. (**a**) Modeling results using the PI model. (**b**) Modeling results using the MPI model. (**c**) Modeling results using the DPI model.

The asymmetric system hysteresis behavior at 1 Hz sinusoidal wave is first modeled using the PI, MPI, and DPI model, respectively with N=25 fundamental operators. Figure 5a shows that using the PI model cannot capture the asymmetric hysteresis behavior accurately and result in a large modeling error. Figure 5b shows that using the MPI model for modeling the system hysteresis is better than the PI model, but the error for the contraction curve is much bigger than that of the expansion curve due to the limitations of the dead zone operators. Although the MPI model achieves slightly better modeling accuracy compared to the PI model, the error is still large for a tremor cancellation handheld surgical instrument such as Itrem [8]. Figure 5c shows that the proposed DPI model performs the best of the three models while describing the asymmetric system hysteresis. The modeling results using three models are summarized in Table 1.

### 3.3. Compensation Results

**Figure 6:**
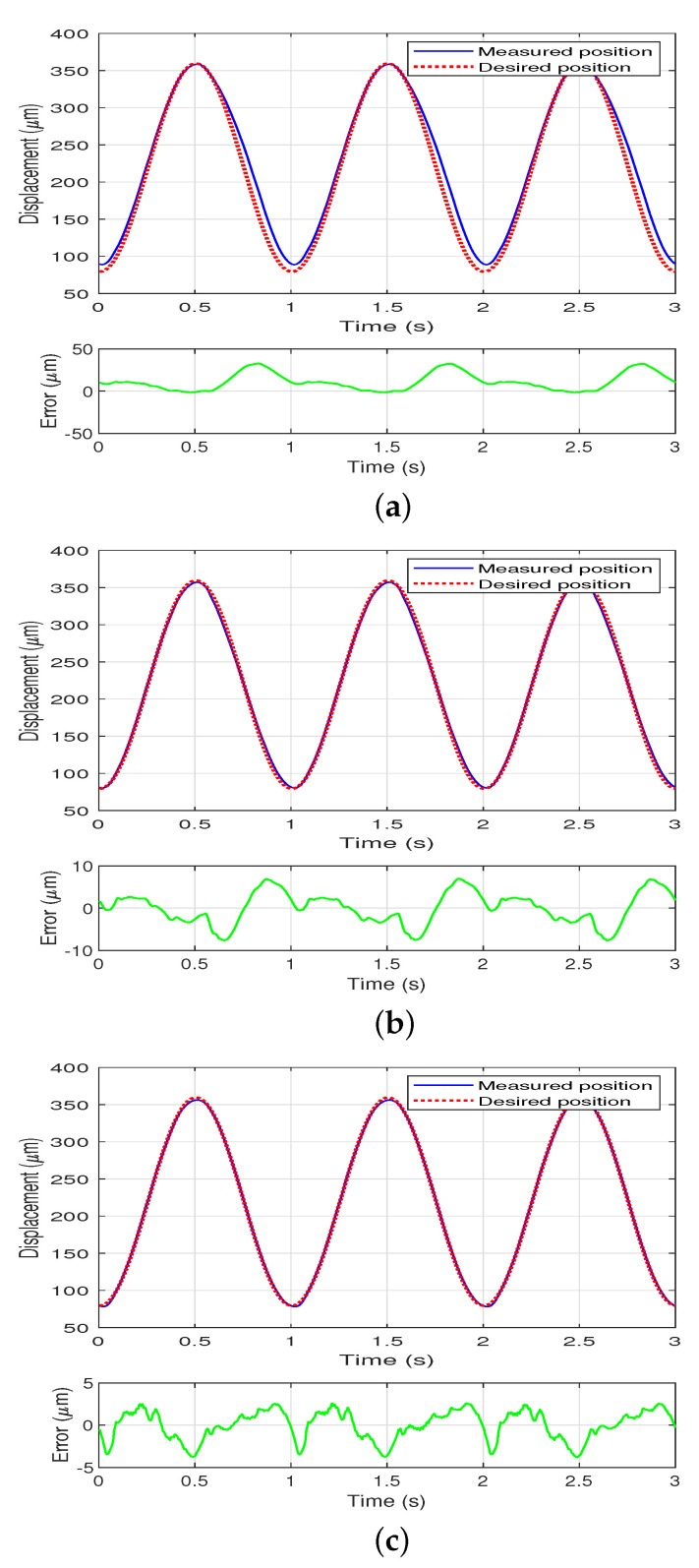
Compensation results at a fixed amplitude using the PI, MPI, and the DPI model, respectively, the dashed line represents the desired position while the solid line represents the measured actual position. (**a**) Compensation results at a fixed amplitude using the PI model. (**b**) Compensation results at a fixed amplitude using the MPI model. (**c**) Compensation results at a fixed amplitude using the DPI model.

To test the performance of the feedforward controller deploying the inverse model, a 1 Hz sinusoidal signal with fixed amplitude $x_{d}\left(t\right)=140\sin\left(2\pi \left(kT_{s}-0.25\right)\right)+219.45$ is used as the control input, where $T_{s}$ is the sampling time with 0.005 s. The response of the system is measured using the PI, MPI, and the DPI model, respectively. The compensation results are shown in Figure 6.

**Figure 7:**
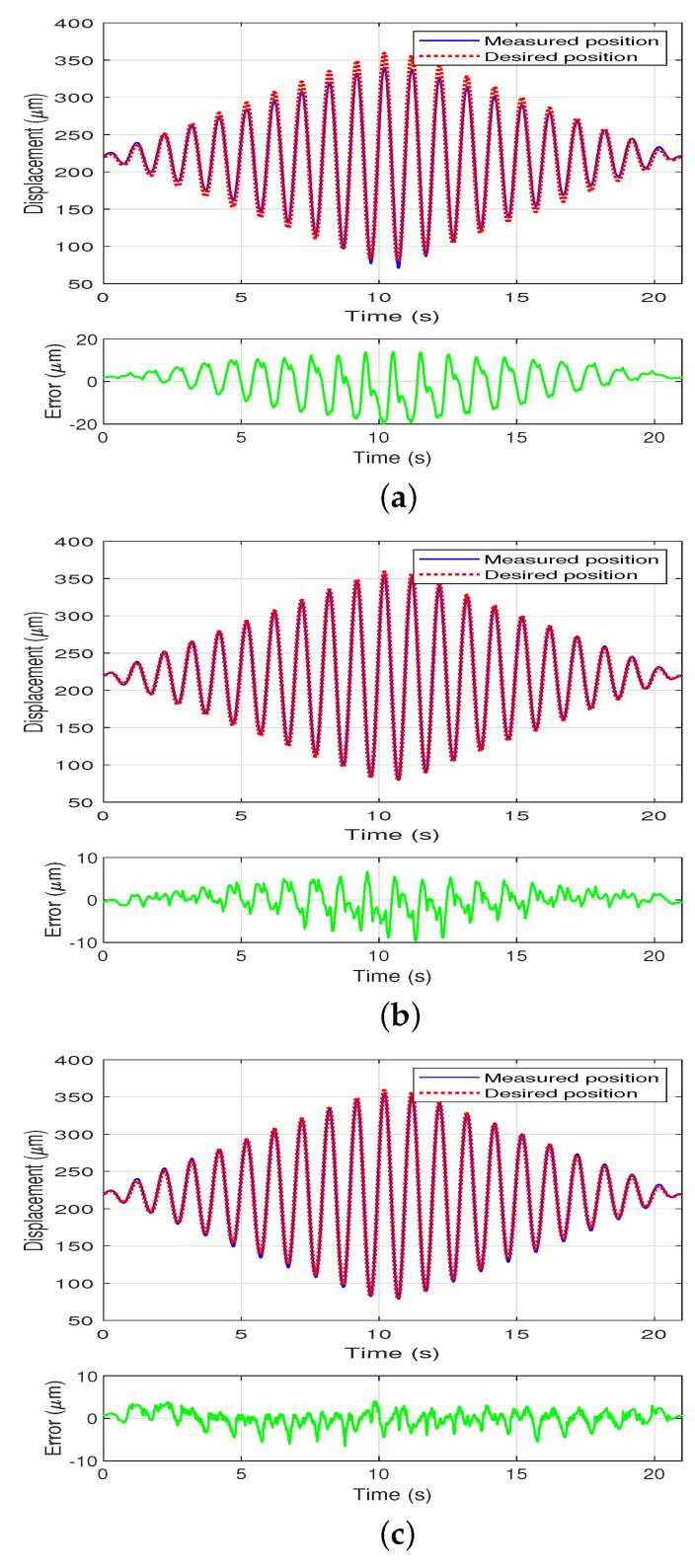
Compensation results at changing amplitudes sinusoidal wave using the PI, MPI and the DPI model, respectively. (**a**) Compensation results at changing amplitudes using the PI model. (**b**) Compensation results at changing amplitudes using the MPI model. (**c**) Compensation results at changing amplitudes using the DPI model.

To further test the performance of the feedforward controller with different magnitudes deploying the inverse model, a 1 Hz sinusoidal signal with gradually changing amplitudes $x_{d}\left(k\right)=A\sin\left(2\pi kT_{s}\right)+219.45$ is used as the control input and the response of the system is measured using the PI, MPI, and the DPI model, respectively. The value of A can be obtained using (33). The compensation results are shown in Figure 7.

(33) $$A=\left\{\begin{array}{c} 14kT_{s},\;k\in Z_{\left[0,2000\right]},\\ 140,\;k\in Z_{\left[2001,2200\right]},\\ 14\left(4200-k\right)T_{s},\;k\in Z_{\left[2201,4200\right]}. \end{array}\right.$$

The compensation results at fixed and varying amplitudes using the PI, MPI, and DPI models are summarized in Table 2. The measured position after compensation is compared to the desired position to calculate the root mean squared error (RMSE). As shown in the table using the PI model to compensate for the asymmetric hysteresis at a fixed amplitude the error is 15.19 μm, while using the MPI model the RMSE is 3.77 μm, with a reduction of 75 percent. Using the DPI model the error can be further reduced to 1.75 μm, with a reduction of 53% compared to the RMSE using the MPI model.

The DPI model also performs the best of the three for compensation of the asymmetric system hysteresis at a changing amplitude, using the DPI model the RMSE is 1.75 μm, with a reduction of 30% compared to the RMSE using the MPI model which is 2.51 μm. The MPI model reduces the error by 66% compared to the RMSE using the PI model which is 7.50 μm. This validates that the proposed DPI model can capture the details of the asymmetric system hysteresis and performs better than the existing MPI model during compensation.

## 4. Discussion

The classical PI model has considerable error for modeling and compensation of asymmetric hysteresis. The dead zone operators are proposed to extend the classical PI model, but the MPI model still has the potential to be improved while modeling the asymmetric system hysteresis. The DPI model is thus proposed to capture the details of the asymmetric system hysteresis by modeling the expansion and the contraction curve respectively. Validation experiments show that the DPI model performs better than the classical PI model and the MPI model, and this proves that the proposed DPI model is effective in modeling and compensating for the asymmetric system hysteresis. The proposed DPI model can reduce the RMSE by around 30% compared to the MPI model, and 76% compared to the PI model.

A similar digitized representation of the MPI model as described in [38] is also applied. With the digitized representation, the inversion calculation can be greatly reduced which may be ideal for some real-time applications.

While implementing the DPI model, the error in percentage at changing amplitudes is slightly bigger than the fixed amplitude, this may be due to the model being sensitive to the rate-dependent hysteresis behavior. Another possible reason is that the distribution used in this paper is a weighted average distribution and a better-weighted strategy may be applied. A training model using the neural networks may also be applied in the future.

The aim of this paper is to propose a generalized model to describe the asymmetric system hysteresis behavior. The DPI model can be further modified for different scenarios, and the backlash can also be included in the model for some applications. The weighted distribution of the observer for updating the fundamental operators may also be further modified to fit different hysteresis behavior.

## 5. Conclusions

A dual-operators based modified Prandtl–Ishlinskii hysteresis model is presented to model and compensate for the asymmetric hysteresis behavior of the piezo-actuated flexure-based system. With a similar digitized representation, the inverse calculation can be greatly simplified. Compared with the MPI model and the PI model, our model yields significantly better experimental results.

## Figures and Tables

**Table 1:** Modeling experimental results.

	RMSE (μm)	RMSE/p-p ampl. (%)	Max Error (μm)
PI	16.08	5.27	38.56
MPI	3.69	1.21	9.73
DPI	0.56	0.18	4.15

**Table 2:** Compensation experimental results.

	RMSE (μm)		RMSE/p-p ampl. (%)		Max Error (μm)	
	**Fixed**	**Changing**	**Fixed**	**Changing**	**Fixed**	**Changing**
PI	15.19	7.50	5.42%	2.70%	32.66	19.90
MPI	3.77	2.51	1.35%	0.90%	7.64	9.74
DPI	1.75	1.76	0.62%	0.63%	3.80	6.56

## Data Availability

Not applicable.
